# Gypsum endemics accumulate excess nutrients in leaves as a potential constitutive strategy to grow in grazed extreme soils

**DOI:** 10.1111/ppl.13738

**Published:** 2022-07-13

**Authors:** Andreu Cera, Gabriel Montserrat‐Martí, Rebecca E. Drenovsky, Alain Ourry, Sophie Brunel‐Muguet, Sara Palacio

**Affiliations:** ^1^ Departamento Biodiversidad y Restauración, Instituto Pirenaico de Ecología Consejo Superior de Investigaciones Científicas Jaca Spain; ^2^ Departament de Biologia Evolutiva, Ecologia i Ciències Ambientals (BEECA), Secció de Botànica i Micologia, Facultat de Biologia Universitat de Barcelona Barcelona Spain; ^3^ Departamento Biodiversidad y Restauración, Instituto Pirenaico de Ecología Consejo Superior de Investigaciones Científicas Zaragoza Spain; ^4^ Biology Department John Carroll University University Heights Ohio USA; ^5^ Agronomie et Nutritions N, C, S, SFR Normandie Végétal (FED 4277), UNICAEN, INRAE, UMR 950 Ecophysiologie Végétale Normandie Université Caen France

## Abstract

Extreme soils often have mineral nutrient imbalances compared to plant nutritional requirements and co‐occur in open areas where grazers thrive. Thus, plants must respond to both constraints, which can affect nutrient concentrations in all plant organs. Gypsum soil provides an excellent model system to study adaptations to extreme soils under current grazing practices as it harbours two groups of plant species that differ in their tolerance to gypsum soils and foliar composition. However, nutrient concentrations in organs other than leaves, and their individual responses to simulated herbivory, are still unknown in gypsum plants. We studied plant biomass, root mass ratio and nutrient partitioning among different organs (leaves, stems, coarse roots, fine roots) in five gypsum endemics and five generalists cultivated in gypsum and calcareous soils and subjected to different levels of simulated browsing. Gypsum endemics tended to have higher elemental concentration in leaves, stems and coarse roots than generalist species in both soil types, whereas both groups tended to show similar high concentrations in fine roots. This behaviour was especially clear with sulphur (S), which is found in excess in gypsum soils, and which endemics accumulated in leaves as sulphate (>50% of S). Moreover, plants subjected to clipping, regardless of their affinity to gypsum, were unable to compensate for biomass losses and showed similar elemental composition to unclipped plants. The accumulation of excess mineral nutrients by endemic species in aboveground organs may be a constitutive nutritional strategy in extreme soils and is potentially playing an anti‐herbivore role in grazed gypsum outcrops.

## 1 INTRODUCTION

The weathering of rocks such as halites, calcites, serpentinites, dolomites or gypsum generates extreme soils with special features restricting plant growth and performance (Kazakou et al., 2008; Moore et al., 2014; Mota et al., 2021; Munns & Tester, 2008; Rorison, 1960). Often, the particular characteristics of extreme soils lead to strong nutrient imbalances in soils, with important consequences for plant nutrition (Lambers & Oliveira, 2019). For example, some elements are frequently found over plant requirements and may become toxic (Kabata‐Pendias, 2010). In contrast, other nutrients are found in low availability, limiting plant growth rates (Aerts & Chapin, 1999). To cope with the strong nutrient imbalances in extreme soils, two main plant nutritional strategies have been suggested in saline, calcicole and metalliferous soils (Lux et al., 2021; Munns & Tester, 2008; Tran et al., 2020). Plants may accumulate excess elements in belowground organs, with roots acting as reservoirs and nutritional barriers, restricting translocation to aboveground parts. Alternatively, plants may move the excess elements to shoots, which requires tolerating high concentrations in aboveground organs.

The nutritional strategies of plants growing in extreme soils have usually been studied in relation to their adaptation to harsh soil conditions (Mota et al., 2017). However, grazing also can influence the ecology of plants adapted to extreme soils since atypical substrates are frequently open areas where herbivores thrive (Pueyo et al., 2008). Herbivore activity can modify soil conditions (Byrnes et al., 2018), increasing the edaphic constraints of extreme environments, such as in serpentine grasslands (Beck et al., 2015), gypsum soils (Moret‐Fernández et al., 2011) and saline soils (Bonis et al., 2005). Plants adapted to extreme soils should have nutritional traits to cope with the harsh soil conditions, but they should also have adaptive traits related to grazing resistance. Plants from frequently grazed ecosystems have evolved with two main response strategies: grazing avoidance or tolerance (Briske & Richards, 1995). These strategies depend on plant growth rates (van der Meijden et al., 1988): plants with rapid growth rates can tolerate grazing, quickly compensating for biomass losses before herbivory reoccurs (Grime, 2006). Compensatory growth traits include increased photosynthetic activity or a rapid re‐translocation of nutrients from roots to shoots (Volenec et al., 1996) when herbivory affects aboveground biomass (Hawkes & Sullivan, 2001). Conversely, plants with slow growth rates are often unable to compensate for biomass loss after herbivory (Grime, 2006) and usually invest strongly in plant defence to avoid consumption (Briske & Richards, 1995). Further, plant responses to grazing can be always expressed (constitutive traits) or only produced in response to grazing events (inductive traits; Moreira et al. 2014). In extreme soils, stress‐tolerant species with slow growth rates dominate over other strategies (Rajakaruna, 2004) because extreme soils are poor in essential nutrients for growth, which may exacerbate herbivory damage due to slow recovery rates (Strauss & Boyd, 2011). Plants growing in extreme soils are more susceptible to herbivory and require significant investment in grazing avoidance mechanisms (Strauss & Cacho, 2013). In turn, high defence costs can lead to trade‐offs in plant competitiveness (Fine et al., 2006), restricting plant distribution to harsh environments and favouring soil specialisation (Fine et al., 2004; Rajakaruna, 2018). Soil specialist species may use unique soil characteristics to acquire defence mechanisms, with plants growing in extreme soils translocating excess nutrients from soil to aboveground parts and improving their defence mechanisms. For example some metal‐hyperaccumulating plants are resistant to herbivory due to their high leaf metal concentrations (Boyd, 2007). However, the extent to which nutrient‐use strategies, adaptation to extreme soils and plant responses to herbivory intersect has been little studied outside metalliferous soils.

Gypsum soils are a suitable model system for studying plant adaptation to extreme soils and the impact of herbivory since gypsum plants have to cope with strong nutrient imbalances and herbivory is a common disturbance in barren gypsum outcrops. Gypsum soils are highly nutrient‐limited, with excess Ca and S and scarce N‐P‐K (Casby‐Horton et al., 2015; FAO, 1990). The high soil Ca and S concentrations far surpass plant nutrient requirements (Merlo et al., 1998) and can become toxic to some plants (Ernst, 1990). Furthermore, gypsum outcrops often support extensive grazing practices (Pueyo et al., 2008). In these harsh environments, two types of gypsum‐adapted species are present, gypsum endemics and gypsum generalists, and they differ in their leaf nutrient composition (Palacio et al., 2007). Gypsum endemic species are mainly restricted to gypsum soils and show high leaf concentrations of elements found in excess in gypsum soils, such as Ca and S (Merlo et al., 2019), often in the forms of sulphate (Ernst, 1990) and gypsum crystals (Palacio et al., 2014). In contrast, gypsum generalist species also appear on soils other than gypsum soils and do not show high leaf nutrient concentrations of Ca or S (Muller et al., 2017). Little is known about nutrient concentrations of gypsum plants in organs other than leaves, such as stems, coarse and fine roots. Such information would help to understand whether gypsum endemics and generalists have different nutritional strategies to cope with the nutrient imbalance of gypsum soils (Mota et al., 2016). For example, gypsum endemics may tolerate excess elements by accumulating them in leaves, whereas gypsum generalists may accumulate excess elements at the root level, preventing nutrient imbalances aboveground.

In addition to being adaptations to harsh soil conditions, these potential and contrasting nutritional strategies between gypsum plants could also be related to grazing resistance. The harsh edaphic and climatic conditions of gypsum environments favour the presence of species with stress tolerance traits (Hodgson et al., 1994), yielding slow growth rates (Grime, 2006). In addition, Braun‐Blanquet and de Bolòs (1958) suggested that gypsum endemic species might be favoured in disturbed gypsum habitats under moderate livestock pressure. Similarly, endemic serpentine taxa tend to occur in bare serpentine microhabitats (Sianta & Kay, 2019). The increased leaf S‐accumulation of gypsum endemics may serve as an anti‐herbivore mechanism (Palacio et al., 2014), as it occurs in metal‐hyperaccumulating plants (Boyd, 2007). S‐rich molecules like glucosinolates in *Brassicales* (Tuominem et al., 2019) or Ca and sulphate crystals in *Acacia* spp. have been suggested to play an anti‐herbivore role (Ernst, 1990; He et al., 2014). However, no previous studies have evaluated the individual responses of gypsum plants to herbivory experimentally, and it is unknown to what extent the atypical foliar elemental concentrations of gypsum endemics (in particular their remarkably high S and sulphate concentrations) are a constitutive or induced trait to avoid being eaten.

The objectives of this study were to investigate the nutritional strategies of gypsum endemic and generalist species in contrasting soil types (gypsum soil vs. calcareous soil, a less ion imbalanced substrate) and evaluate the response of these plants to simulated browsing (removal of 66% aerial biomass vs. unclipped control). We analysed whole plant partitioning of elements among different organs (leaves, stems, coarse roots and fine roots) and sulphate accumulation in leaves. In addition, we analysed plant biomass and root mass ratio to assess plant performance and growth strategies. We hypothesised that: (1) Gypsum endemic and generalist species would show differences in elemental partitioning among organs and in leaf sulphate accumulation. Gypsum endemics would accumulate elements found in excess on gypsum soils (S, Ca) across the plant, but especially sulphate in leaves, whereas generalists would accumulate excess elements in fine roots as a nutritional barrier to avoid toxicity. (2) The prevalence of gypsum‐adapted species in grazed areas is due to avoidance rather than tolerance mechanisms because they are stress‐tolerant species with slow growth rates. Consequently, we expected they would not be able to compensate for the biomass lost due to clipping following 1 year of growth. (3) However, gypsum endemics would respond to clipping by increasing the concentration of total S and sulphate in leaves, as an induced mechanism of grazing deterrence, a strategy that we did not expect to find in generalist species.

## 2 MATERIALS AND METHODS

### 2.1 Study species

We selected taxa based on their ecological significance in gypsum communities, similar in growth form, and, where possible, with phylogenetic relationships. All of the gypsum endemic and generalist taxa selected are dominant subshrubs in gypsum ecosystems of north‐eastern Spain, and one pair represent congeners and three taxa represent confamilials. All gypsum endemic species selected show a high affinity for gypsum soils in Spain (Mota et al., 2011). Gypsum endemics included *Gypsophila struthium* subsp. *hispanica* (Willk.) G. López. (Caryophyllaceae), *Herniaria fruticosa* L. (Caryophyllaceae), *Helianthemum squamatum* Pers. (Cistaceae), *Lepidium subulatum* L. (Brassicaceae), *Ononis tridentata* L. (Fabaceae); and generalist species were *Boleum asperum* Desv. (Brassicaceae), *Helianthemum syriacum* (Jacq.) Dum.Cours.(Cistaceae), *Linum suffruticosum* DC.(Linaceae), *Matthiola fruticulosa* (L.) Maire (Brassicaceae) and *Rosmarinus officinalis* L. (Lamiaceae).

### 2.2 Experimental design

For each taxon, seeds were collected from several individuals within the population growing in gypsum soils and stored in paper bags. Field soils were collected in unfertilized areas to 50 cm depth, removing the O horizons prior to sampling. Then, soils were sieved to 1 cm and homogenised before being used to fill the pots. Gypsum soil was collected in the Middle Ebro Basin (41°41′44.5″ N, 0°44′26.7″ W) and calcareous soil was collected from the Iberian System (41°30′45.8″N, 1°26′47.8″W). Plants were grown from seeds in 0.06‐L square pots in April 2016. Half of the pots contained calcareous native soil and the other half contained gypsum native soil (see Cera, Montserrat‐Martí, et al., 2021 for further details). Seven months after emergence (November 2016), plants were transplanted into 7‐L square pots (large species) and 5.6‐L square pots (small species). Five months after transplantation, the plants were thinned to leave one individual per pot. Clipping treatments were applied in October 2017: five replicates of each species and soil combination were clipped and five were left unclipped as controls. The clipping treatment consisted of removing 66% of shoots with secateurs, leaving the apical stem undamaged and applying the same proportion of leaf area removal to all replicates within a species. Plants were kept well‐watered throughout the experiment by regular watering with tap water until soils were saturated. There were no trays under pots, allowing the soils to drain to field capacity. Plants were moved to a greenhouse between November and March to avoid freezing. All plants were harvested between September and November 2018, 1 year after clipping.

### 2.3 Plant biomass

At harvest, plants were separated into their main organs: leaves, stems, coarse roots and fine roots (<2 mm in diameter). We cut off the aboveground parts, separating green leaves, senescent leaves and stems. Next, pots were emptied and all roots were separated from the soil using tweezers. Fine roots were separated from coarse roots, selecting those that were less than 2 mm in diameter (Pérez‐Harguindeguy et al., 2016). All clipped and harvested material was rinsed with tap water and dried in an oven at 50°C for 5 days. All dry plant fractions were weighed on a precision scale (42 g/0.00001 g, MS105DU, Mettler Toledo).

### 2.4 Elemental analyses

All dried organs were finely ground using a ball mill (Retsch MM200, Restch GmbH). Nitrogen (N) and carbon (C) concentrations were analysed with an elemental analyser (TruSpec CN, LECO). The elemental concentrations of Al, As, Ca, Cd, Co, Cr, Cu, Fe, Hg, K, Li, Mg, Mn, Mo, Na, Ni, P, Pb, S, Se, Si, Ti, V, Zn were measured by extracting samples with HNO_3_‐H_2_O_2_ (8:2) by microwave acid digestion (Speed Ave MWS‐3+, BERGHOF), followed by inductively coupled plasma‐optical emission spectrometry (Varian ICP 720‐ES, Agilent Technologies). All elemental analyses were performed by EEZ‐CSIC Analytical Services.

### 2.5 Sulphate extraction and quantification

About 20 mg of ground leaf dry matter was used in a four‐step extraction process. Leaf material was mixed with 0.5 ml of 50% ethanol and then incubated at 45°C for 1 h. After centrifuging the mixture for 10 min at 10,000*g* (4°C), the supernatant was collected and the pellet was re‐extracted following the same procedure. The last two extractions were performed on the pellet with 0.5 ml distilled water at 95°C for 1 h (for each extraction). The final 2 ml of supernatant was concentrated for 20 hours at room temperature (Concentrator plus, Vacufuge® plus, Eppendorf), then resuspended in 1 ml of ultrapure water and filtered (0.5 μm). The sulphate concentration was determined by High Liquid Performance Chromatography (HPLC, HPLC, DX100, Dionex Corp.) as described in Akmouche et al. (2019).

### 2.6 Calculations and statistics

All statistical and graphical analyses were carried out using R version 4.0.2 (R Core Team, 2022). The graphs were designed with ggplot2 package 3.3.1 (Wickham, 2009). Packages used for each statistical analyses are specified later.

Treatment effects on plant growth were assessed by analysing differences in canopy height, plant biomass and root mass ratio by generalised linear mixed models (GLMM) in lme4 package version 1.1‐23 (Bates et al., 2007). Differences in growth variables were modelled with soil type, gypsum affinity and clipping treatment as fixed factors, species as random factor and preclipping plant dimensions as a covariate. This covariate was the first component of a Principal Component Analysis (PCA) including canopy area, canopy height and canopy length of plants before the clipping treatment. Including this covariate in linear models accounted for variation between individuals due to their initial size and morphology and not treatments (Palacio et al., 2008). PCA was performed using the *rda* function in the vegan package (Oksanen et al., 2007). We analysed intraspecific differences between treatments within each species using generalised linear models (GLM). In all models, Shapiro–Wilk and Bartlett's K‐squared tests were performed to check for normality and homoscedasticity of residuals. When there was not a normal distribution of residuals, models were fitted to a negative binomial or a gamma distribution, according to the lower AIC. Significance of differences was evaluated using a Wald test with the *Anova* function in the car package (Fox & Weisberg, 2019). When differences were statistically significant, multiple comparisons between the levels of each factor or interaction of factors were assessed with the *glht* function in the multcomp package version 1.4‐13 in R (Hothorn et al., 2009).

Effects on plant nutrition were analysed using the elemental concentrations and pools of different organs. Elemental concentrations were mass‐based concentrations of the different elements analysed, whereas elemental pools were calculated as a percentage of the total plant biomass by multiplying the mass‐based concentrations of the different elements in each organ by its biomass and dividing by total plant biomass. We described elemental nutrition using one‐dimension data (the concentration of each element and sulphate) and multidimensional data (the composition of concentrations, excluding sulphate data). Compositional data was a vector of all elements for each replicate, transformed to Center Log Ratio to avoid scale invariance (Aitchison, 1982; Soriano‐Disla et al., 2013) with the composition package version 1.40‐5 (van den Boogaart & Tolosana‐Delgado, 2008).

We assessed differences among soil type, gypsum affinity and clipping treatment for both compositional and one‐dimensional elemental concentration data. Differences in one‐dimensional data were assessed using GLMMs. We included taxonomic family and species nested within family as random factors. Models were fitted to a Gamma distribution when there was not a normal distribution of residuals since, in most cases, data had a constant coefficient of variation and variances increased with means (McCullagh & Nelder, 1989). Model link functions of the Gamma distribution were selected according to the lower AIC criterion. When differences were statistically significant, multiple comparisons among levels of each factor or interaction of factors were assessed. Differences in compositional data were assessed using PERMANOVA with Euclidean distances using the *adonis* function in the vegan package. Similarly, intraspecific differences between soil types and clipping treatment for each species were assessed for elemental concentrations of one‐dimensional data and compositional data. These models included clipping treatment and soil type as fixed factors and preclipping plant dimensions as a covariate. We performed a PCA with the compositional data of elemental concentrations to analyse the relationships among treatments and soil composition; the procedure followed was the same as described above for the PCA of pretreatment size variables.

## 3 RESULTS

### 3.1 Substrate and clipping effects on the elemental concentrations across plant organs of gypsum endemic and generalist species

The multivariate elemental composition of plants differed between organs, between endemic and generalist species, and between plants grown on gypsum versus calcareous soils (*p* < 0.05, Table 1). However, clipping did not alter the elemental composition of plants. From a compositional perspective, the elemental concentrations in plant organs clearly differed from that of the soil (Figure S1). Generally, leaves and fine roots were the organs with the highest concentrations of total S, Ca, Mg, Al, Fe, whereas coarse roots showed generally higher concentrations of total K, P and Zn (*p* < 0.05, Figure S1). Furthermore, all organs showed different elemental compositions depending on whether plants were grown on gypsum (generally higher concentrations of total S, Mg and Ca) or on calcareous soils (typically higher concentrations of total K and P). Likewise, there were different patterns in endemics and generalists: endemics showed higher concentrations of total S, Mg and Ca. Elemental concentrations in leaves, stems and coarse roots were more similar among plants in the same gypsum affinity group than in plants cultivated on the same soil type, whereas fine roots were more similar between plants grown on similar soils, independent of gypsum affinity (Figure S1).

**TABLE 1:** PERMANOVA testing the effect of organ, affinity to gypsum soils, soil type, clipping and their interaction on the elemental composition of plants

	F‐ratio	*p*‐Value
Organ	**75.76**	**0.001**
Gypsum affinity	**35.28**	**0.001**
Soil type	**31.45**	**0.001**
Clipping	0.34	0.817
Organ × Gyp. aff.	**9.59**	**0.001**
Organ × Soil	**5.70**	**0.001**
Gyp. aff. × Soil	0.84	0.365
Organ × Clip.	0.11	1.000
Gyp. aff. × Clip.	0.45	0.713
Soil × Clipping	0.21	0.952
Organ × Gyp. × Soil	0.42	0.923
Organ × Gyp. × Clip.	0.08	1.000
Organ × Soil × Clip.	0.08	1.000
Gyp. × Soil × Clip.	0.26	0.928
Organ × Gyp. × Soil × Clip.	0.13	1.000

*Notes*: F‐ratios and *p*‐values are shown. Bold type indicates significant effects.

**FIGURE 1:**
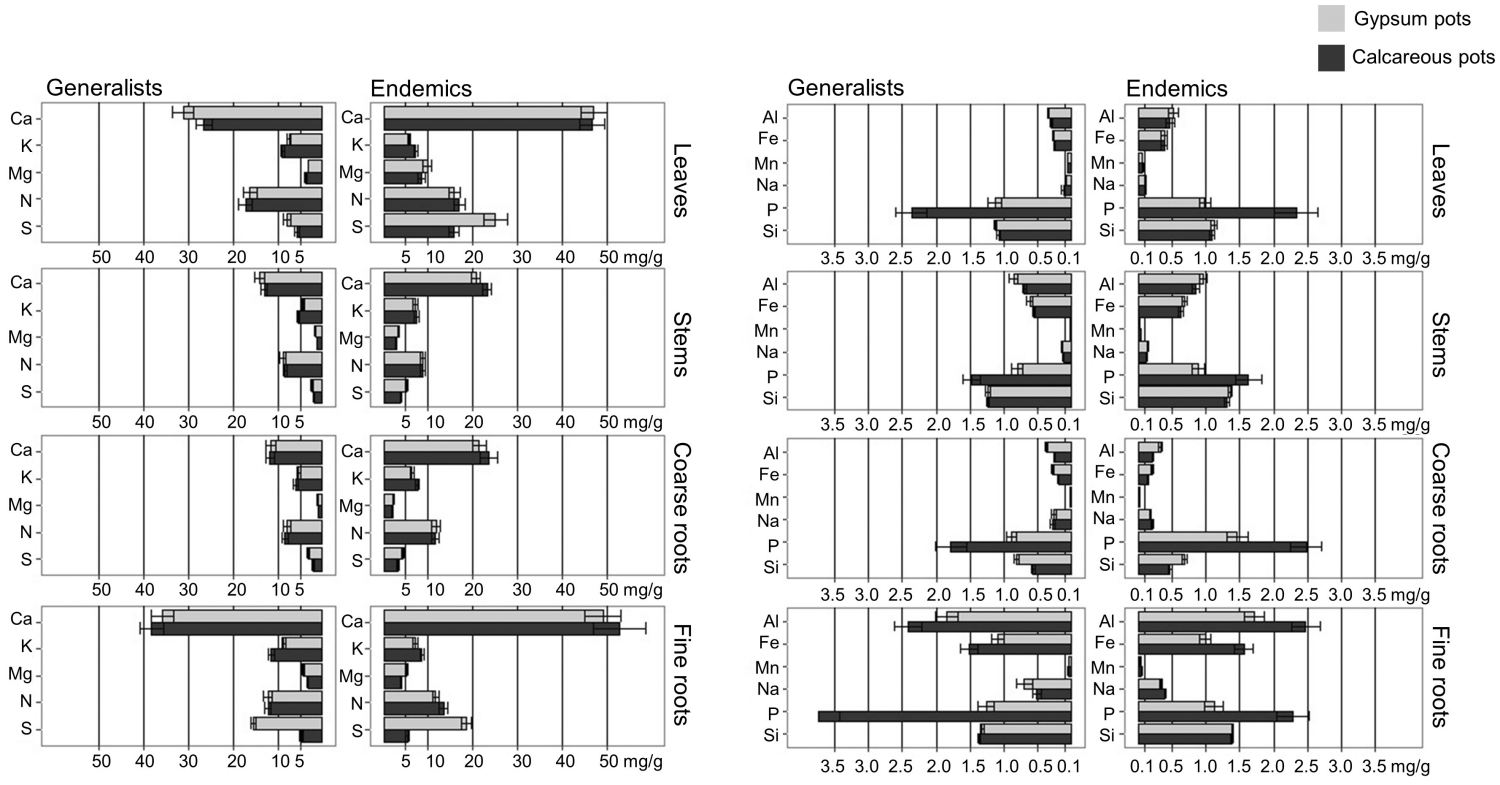
Barplots of elemental concentration in each organ in gypsum and calcareous pots between generalist and endemic species. Means and SE are represented (*n* = 185 leaf samples, *n* = 198 stem samples, *n* = 173 coarse root samples, *n* = 198 fine root samples)

Analysing the concentrations of individual elements, plants grown on gypsum soils tended to show higher total S and lower P concentrations in all organs than when grown on calcareous soils (Figure 1; see anova results in Table S1 and means and SEs in Table S2). Additionally, differences among elements between endemic and generalist plants depended on organ type. Endemic species tended to have higher leaf, stem, and coarse root nutrient concentrations for many elements compared to generalist species, but this trend did not hold in fine roots. Clipping did not affect the concentrations of individual elements, and no significant interactions were observed between clipping and the other factors analysed. However, there was a significant interaction among soil type, gypsum affinity and organ for the concentrations of Na, P and S. For example, leaf, stem and coarse root total S concentrations were highest in endemics grown on gypsum, followed by endemics grown on calcareous soil, then generalists grown on gypsum, and finally generalists grown on calcareous soil. In fine roots, the highest total S concentrations were found in plants grown on gypsum, and the lowest, in plants grown in calcareous pots, irrespective of their affinity for gypsum soils. In addition, in fine roots, generalists showed higher P and Na than endemics, especially in calcareous soils for P. Some species showed different responses to these general trends in the elemental concentrations of certain elements between clipping and soil treatments (see anova results in Table S3 and means and SEs in Table S4).

### 3.2 Sulphate accumulation in leaves

**FIGURE 2:**
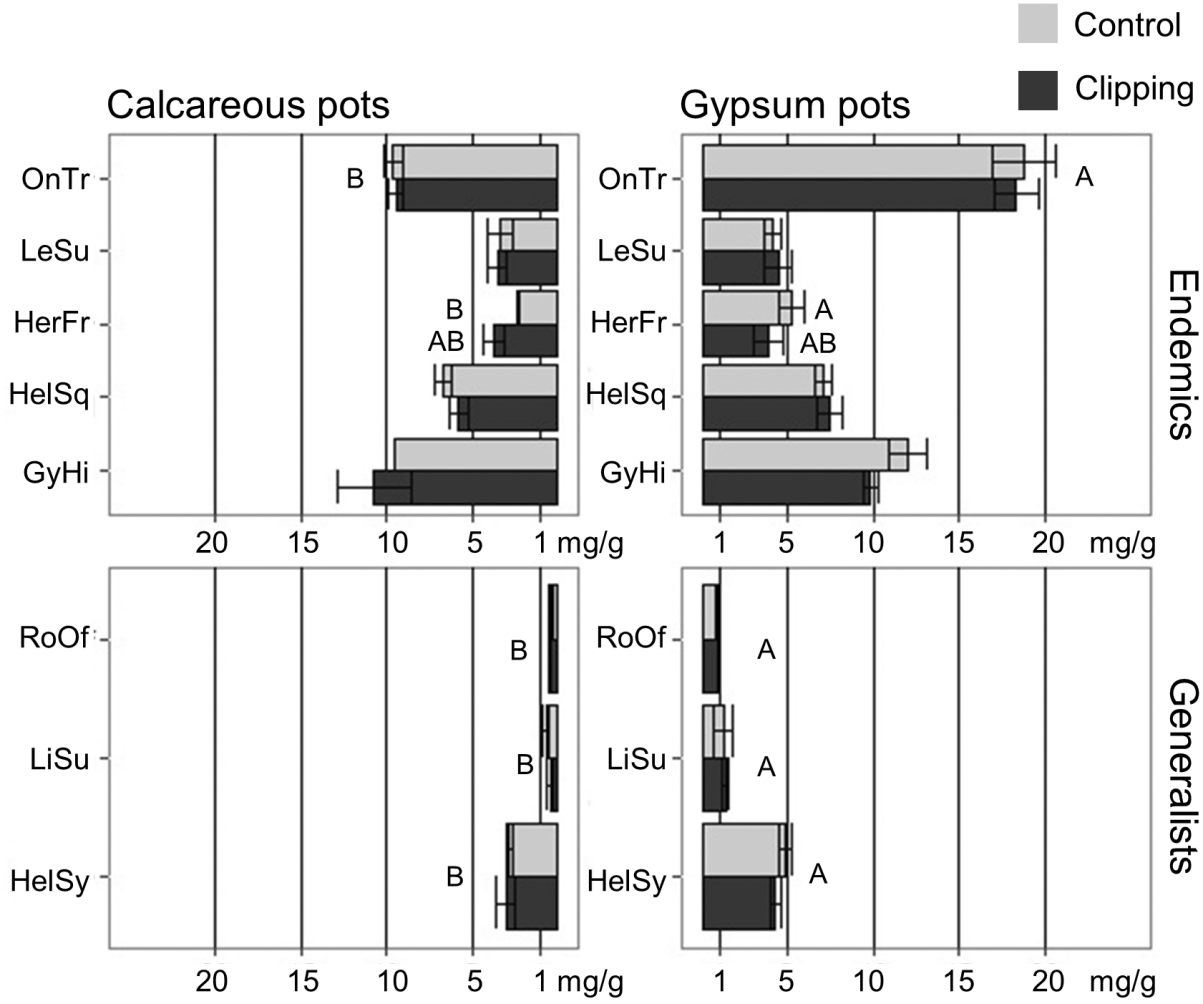
Barplots of sulphate accumulation in leaves in study species. Means and SE are represented (*n* = 94 leaf samples). Significant *p*‐values of treatments are shown. Letters indicate significant differences between treatments after multiple comparison tests in each species. OnTr, *Ononis tridentata*, LeSu, *Lepidium subulatum*; HerFr, *Herniaria fruticosa*; HelSq, *Helianthemum squamatum*; GyHi, *Gypsophila hispanica*

**FIGURE 3:**
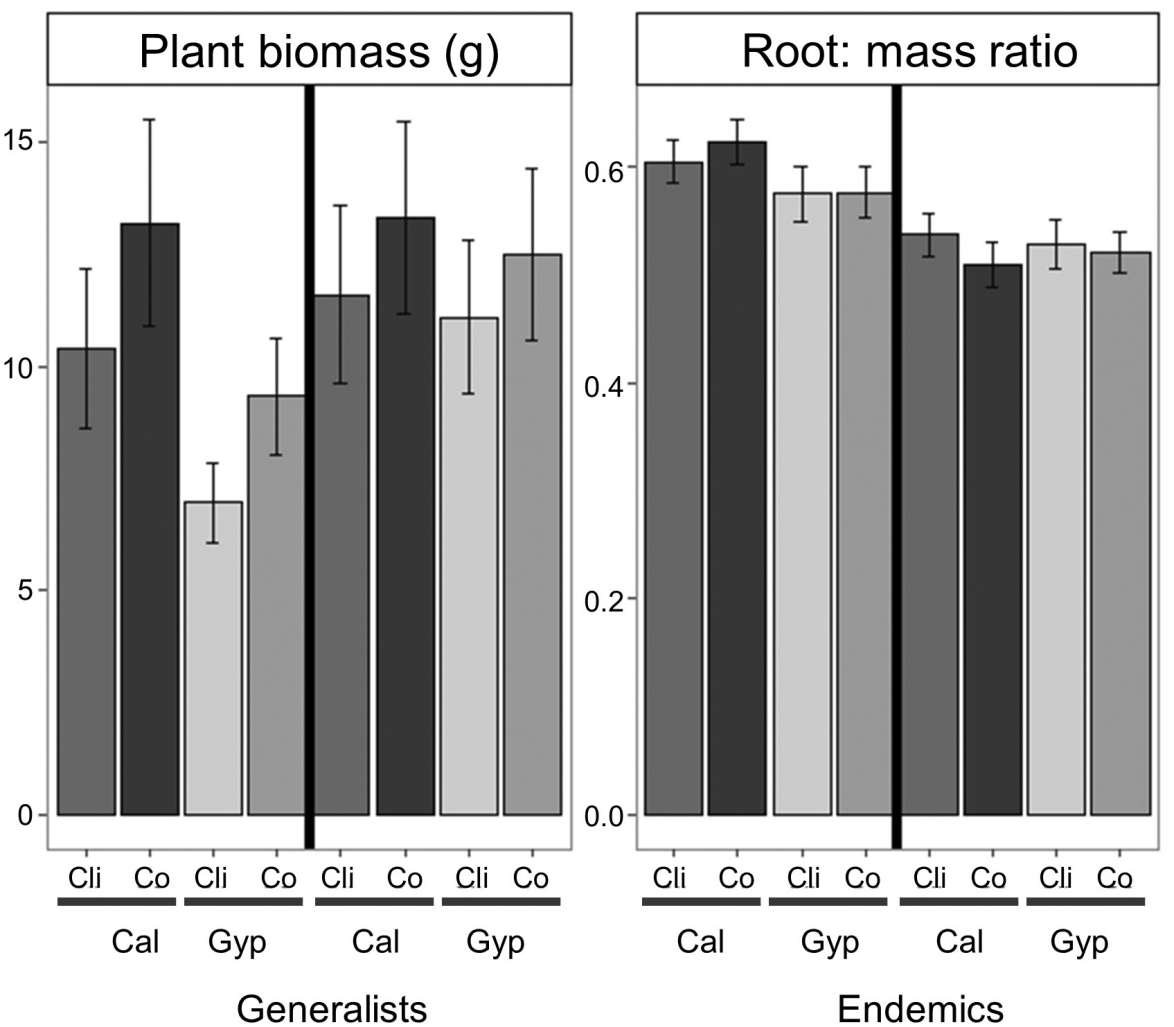
Barplots of plant biomass and root mass ratio of control and clipped gypsum plants in calcareous and gypsum pots. Means and SE are represented (*n* = 197 plants). Cal were plants grown in calcareous soils. Gyp were plants grown in gypsum soils. Cli were clipped plants. Co were control plants

Sulphate accumulation (Figure 2) differed between plants grown on gypsum and calcareous soils (Chisq: 41.16, *p* < 0.05). However, clipping did not alter plant sulphate accumulation (Chisq: 0.01, *p* = 0.917), and the interaction between soil and clipping also was not significant (Chisq: 0.38, *p* = 0.537). Plants grown in gypsum soil had higher sulphate in leaves than those grown in calcareous soil, although species showed different degrees of sulphate concentration. Gypsum endemics were characterised by higher leaf sulphate accumulation whatever the soil type (Figure 2) and endemic species such as *G. hispanica*, *H. squamatum* and *O. tridentata* had high leaf sulphate (above 5 mg/g, Figure 3), whereas generalists such as *L. suffruticosum* and *R. officinalis* had low leaf sulphate (below 1 mg/g, Figure 3).

### 3.3 Effects on plant growth and biomass allocation

Clipped plants accumulated less biomass than control plants 1 year after clipping (*p* < 0.05, Figure 3, Table S5). However, clipping did not affect the root mass ratio of plants. Soil type also significantly affected plant growth: plants grown in calcareous soil showed higher biomass than those grown on gypsum (*p* < 0.05). In addition, the interaction between soil type and gypsum affinity was the only significant interaction found (*p* < 0.05). Generalists grown on calcareous soils accumulated more biomass than those grown on gypsum soil, whereas gypsum endemics showed no difference in biomass between soil treatments. The interaction among clipping, gypsum affinity and substrate was not significant, indicating that, contrary to our expectation, gypsum endemics and generalists showed comparable responses to clipping on both substrates. However, the responses of certain species differed from these general trends (Table S6).

## 4 DISCUSSION

In accordance with our expectations, gypsum endemic species had a higher concentration of the elements found in excess in gypsum soils in their leaves, stems and coarse roots than generalist species. However, in contrast to our first hypothesis, endemics and generalists had similar concentrations in fine roots. In support of our second hypothesis, endemics and generalists were unable to compensate for biomass losses in any soil after clipping. Contrary to our third hypothesis, endemics, like generalists, did not respond to clipping by increasing either total S or sulphate concentration in leaves.

### 4.1 Gypsum endemics accumulated elements found in excess in soils in leaves, stems and coarse roots, whereas generalists did not

Plant affinity for gypsum soils is related to a particular elemental composition. Similar to previous studies, gypsum endemics had higher leaf S and sometimes higher Ca and Mg concentrations than generalist species (Merlo et al., 2019; Muller et al., 2017; Palacio et al. 2007, 2022). This singular elemental composition is a constitutive nutritional strategy of gypsum endemics regardless of whether they grow in calcareous or gypsum soils (Cera, Montserrat‐Martí, et al., 2021). Furthermore, our results are the first experimental evidence that generalists and endemics differ in their elemental composition across organs. Gypsum endemics tended to have a higher elemental concentration in leaves, stems and coarse roots than generalists, whereas both groups had similar concentrations in fine roots. This behaviour was especially clear with S, the most discriminating element between calcareous and gypsum soils (Cera, Montserrat‐Martí, et al., 2021) and between gypsum endemics and generalists for foliar concentrations (Merlo et al., 2019). Plants adapt to excess elements in soils (in our case S) by accumulating them in roots, as a nutritional barrier, or by tolerating them in leaves (Tran et al., 2020). Gypsum endemic species are hence leaf accumulators, whereas generalists seem to use mechanisms that block S uptake at the fine root level, similar to other soil endemics and generalists in extreme soils. For example, halophytes accumulate higher foliar Na and Cl concentrations than glycophytes when growing in saline soils (Matinzadeh et al., 2019; Munns & Tester, 2008), and calcifuge plants have apoplasmic barriers in roots to prevent excess Ca in leaves (Lux et al., 2021), whereas calcicole plants tolerate high Ca concentrations in leaves (White & Broadley, 2003).

The differential nutritional strategy between gypsum endemics and generalists may influence plant nutrition and, ultimately, plant performance on gypsum soils. Generalist species may block excess elements in roots (especially cations), probably developing apoplastic barriers in the endodermis (Sattelmacher, 2001), such as Casparian band strips and suberin lamellae formation, similar to plants in saline, calcareous, and metalliferous environments (Barberon, 2017; Lux et al., 2021; White & Broadley, 2003). Endodermal barriers reduce the permeability of elements from the rhizosphere to the plant, leading to decreases in foliar concentrations of Ca, Mn and Zn (Courbet et al., 2019). Blocking S at the root level in generalists may be achieved by decreased expression of S transporters since sulphate uptake predominantly follows the symplastic route (Hawkesford et al., 2012) and because aboveground organs may have low demand in contrast to high soil concentrations (Davidian & Kopriva, 2010; Lappartient & Touraine, 1996). Such reduced S uptake may in turn interfere with other nutrients such as Mo and Se, which can also share transporters with sulphate (Courbet et al., 2019), although we could not detect Mo and Se with ICP‐OES.

In contrast, gypsum endemics may be more permeable to nutrients in general, through reduced apoplastic barriers, and likely enhanced symplastic uptake through regulated expression of sulphate transporters (Davidian & Kopriva, 2010), particularly when grown in soils with lower S availability, like calcareous soils. Similarly, the S accumulator *Brassica napa* upregulated the expression of sulfate transporters when cultivated on low S media (Koralewska et al., 2007). Gypsum is considered a very nutrient‐limited soil, especially for N, P, K, Fe and some micronutrients (FAO, 1990). If gypsum endemics show fewer endodermal barriers and improved S uptake, they could be more efficient in the uptake of these scarce nutrients. Nevertheless, we did not observe better growth on gypsum soils by endemics relative to generalists or better accumulation of N and P in leaves. Indeed, generalists showed a higher accumulation of P in fine roots. This was probably because they have more root colonisation by arbuscular mycorrhiza fungi than endemics (Cera, Duplat, et al., 2021). More research on nutrient acquisition and growth of gypsum‐adapted species is needed to fully understand the implications of the different nutritional strategies of gypsum endemics and generalists.

Gypsum endemic species are sulphate accumulators in leaves. Such capacity to store S mostly as sulphate has been shown in cultivated Brassicaceae such as *Brassica napus* (in which S‐sulphate may account for up to 70% of leaf S; Sarda et al., 2014). In this species, sulphate was involved in vacuolar S storage, a pool for remobilisation when S soil availability decreases (Abdallah et al., 2010), but it also acted as a significant contributor to leaf osmotic potential (Sorin et al., 2015). Endemic species could maintain their capacity to accumulate sulphate in leaves for storage or osmotic purposes even when grown in calcareous soil, probably by upregulating the expression of root sulphate transporters, as previously reported for plants facing S deficiency (Abdallah et al., 2010). Additionally, gypsum endemic plants can accumulate high concentrations of sulphate with calcium in leaf vacuoles (Ernst, 1990; Kinzel, 1989) via elemental biomineralisation of gypsum crystals (He et al., 2014). *Lepidium subulatum*, an endemic Brassicaceae species, did not show a high accumulation of sulphate as it can accumulate other S‐rich organic compounds like glucosinolates (Tuominem et al., 2019). In addition to the putative osmotic role, S‐accumulation, either as biocrystals or as S‐rich organic compounds, can also play an anti‐herbivore role (Ernst, 1990; He et al., 2014), with potential implications for the grazing resistance of gypsum endemics.

### 4.2 Plants were unable to compensate for biomass losses in any soil after clipping

As predicted, plants were negatively affected by clipping irrespective of soil type and gypsum affinity. One year after clipping, all species accumulated less biomass in clipped compared to control plants. Further, none of the studied species showed higher leaf N in clipped plants, which would have been an indicator of higher leaf activity and a trait related to grazing tolerance (Capó et al., 2021). Compensation varies depending on resource availability and how well adapted plants are to low or high resource availability because resource availability ultimately affects growth rates (Wise & Abrahamson, 2005). Gypsum endemics seem to have evolved under grazing pressure, as gypsum plant communities are usually shrublands or grasslands (Mota et al., 2017), community types associated with large wild mammals and livestock grazing (Asner & Levick, 2012; Bakker et al., 2016). Our results support the notion that gypsum endemics are stress‐tolerant plants with low growth rates. Contrary to generalist species, which grew better on calcareous than gypsum soils, they maintained similar growth rates in both substrates even though they had higher nutrient concentrations in calcareous soils (Cera, Montserrat‐Martí, et al., 2021). The slow growth strategy of gypsum endemics implies a disturbance avoidance, rather than tolerance strategy (Grime, 2006), with high investments in plant defence rather than increased growth rates to compensate for biomass loss after consumption (Strauss & Cacho, 2013). The lower ability of gypsum endemics to increase their growth rate on calcareous soils may also explain why these species are outcompeted by faster‐growing tolerant species of gypsum (Sianta & Kay, 2019).

### 4.3 Grazing did not alter S accumulation in gypsum endemics

Previous studies have suggested gypsum endemics are favoured under moderate livestock grazing pressure (Braun‐Blanquet & de Bolòs, 1958) due to foliar accumulation of gypsum crystals (Palacio et al., 2014). Consequently, we hypothesised that S‐accumulation could be an induced mechanism in response to grazing. However, clipping did not alter the elemental composition or S‐accumulation of studied plants. This could be explained by our short‐term experiment in which a single clipping event might have been insufficient to induce modification of S‐accumulation in contrast to longer‐term experiments, where repeated and sustained grazing is required to induce a response in plants (Canadell & López‐Soria, 1998). Alternatively, grazing could filter plants at the population level with constitutively higher leaf S concentration rather than producing an induced mechanism (Bolnick et al., 2011).

## 5 CONCLUSIONS

Gypsum endemic and generalist species showed differences in elemental partitioning across organs and sulphate accumulation in leaves. Accumulating excess nutrients found in gypsum soils by endemic species is a constitutive nutritional strategy comparable to that of endemics in saline, calcareous, metalliferous and gypsum soils. Gypsum is a nutrient‐limited soil, especially for phosphorus, which imposes restrictions on plant growth. The unique nutritional strategy would be an ecological advantage as soil specialists, but more studies that combine experimental and field approaches are needed to elucidate if this strategy plays an anti‐herbivore role, enhances nutrient acquisition, or plays another potential role, such as osmotic adjustment, in this harsh environment common in drylands.

## AUTHOR CONTRIBUTIONS

Gabriel Montserrat‐Martí and Sara Palacio designed and set up the experiment; Andreu Cera, maintained the experiment, measured all variables, analysed data and led the manuscript writing; Sophie Brunel‐Muguet measured sulphate; All authors discussed the results and wrote the manuscript.

## FUNDING INFORMATION

This work was supported by Gobierno de España [MICINN, CGL2015‐71360‐P and PID2019‐111159GB‐C31]; by European Union's Horizon 2020 [H2020‐MSCA‐RISE‐777803]. AC and SP were funded by a FPI fellowship [MICINN, BES‐2016‐076455] and a Ramón y Cajal Fellowship [MICINN, RYC‐2013‐14164], respectively.

## Supporting information


**Appendix S1** Supporting Information

## Data Availability

Data that support the findings of this study are available upon reasonable request from the corresponding author.
